# Identification of Five Cytotoxicity-Related Genes Involved in the Progression of Triple-Negative Breast Cancer

**DOI:** 10.3389/fgene.2021.723477

**Published:** 2022-01-03

**Authors:** Yan Zhang, Gui-hui Tong, Xu-Xuan Wei, Hai-yang Chen, Tian Liang, Hong-Ping Tang, Chuan-An Wu, Guo-Ming Wen, Wei-Kang Yang, Li Liang, Hong Shen

**Affiliations:** ^1^ Department of Pathology, School of Basic Medical Sciences, Southern Medical University/Nanfang Hospital, Southern Medical University, Guangzhou, China; ^2^ Department of Pathology, The First Affiliated Hospital of Guangdong University Of Pharmacy, Guangzhou, China; ^3^ Department of Pathology, Shenzhen Longhua District Maternity & Child Healthcare Hospital, Shenzhen, China; ^4^ Department of Pathology,The first Affiliated Hospital,Guangzhou Medical University, Guangzhou, China; ^5^ Department of Pathology, Shenzhen Maternity & Child Healthcare Hospital, Shenzhen, China; ^6^ Department of Prevention and Health Care, Shenzhen Longhua District Maternity & Child Healthcare Hospital, Shenzhen, China; ^7^ Guangdong Province Key Laboratory of Molecular Tumor Pathology, Guangzhou, China

**Keywords:** DUOX2, CD8^+^ T cell, GALNTL5, breast cancer, therapeutic efficacy

## Abstract

**Background:** Breast cancer is one of the deadly tumors in women, and its incidence continues to increase. This study aimed to identify novel therapeutic molecules using RNA sequencing (RNA-seq) data of breast cancer from our hospital.

**Methods:** 30 pairs of human breast cancer tissue and matched normal tissue were collected and RNA sequenced in our hospital. Differentially expressed genes (DEGs) were calculated with raw data by the R package “edgeR”, and functionally annotated using R package “clusterProfiler”. Tumor-infiltrating immune cells (TIICs) were estimated using a website tool TIMER 2.0. Effects of key genes on therapeutic efficacy were analyzed using RNA-seq data and drug sensitivity data from two databases: the Cancer Cell Line Encyclopedia (CCLE) and the Cancer Therapeutics Response Portal (CTRP).

**Results:** There were 2,953 DEGs between cancerous and matched normal tissue, as well as 975 DEGs between primary breast cancer and metastatic breast cancer. These genes were primarily enriched in PI3K-Akt signaling pathway, calcium signaling pathway, cAMP signaling pathway, and cell cycle. Notably, CD8^+^ T cell, M0 macrophage, M1 macrophage, regulatory T cell and follicular helper T cell were significantly elevated in cancerous tissue as compared with matched normal tissue. Eventually, we found five genes (*GALNTL5, MLIP, HMCN2, LRRN4CL,* and *DUOX2*) were markedly corelated with CD8^+^ T cell infiltration and cytotoxicity, and associated with therapeutic response.

**Conclusion:** We found five key genes associated with tumor progression, CD8^+^ T cell and therapeutic efficacy. The findings would provide potential molecular targets for the treatment of breast cancer.

## Introduction

Breast cancer is a deadliest type of female carcinoma (Xu et al., 2021), the incidence of breast cancer is increasing in the past few years (Hu et al., 2021; Su et al., 2021; Tagliamento et al., 2021; Xu et al., 2021). Although the treatment of breast cancer has achieved great progress (Li et al., 2021; Mouabbi et al., 2021), 5-years survival rate for advanced breast cancer is still poor (Kang et al., 2018; Wang et al., 2021a). Accordingly, the molecular mechanisms of breast cancer occurrence and progression are still largely unclear.

Triple-negative breast cancer (TNBC) is an aggressive and heterogeneous subtype of breast cancer. The characteristics of TNBC on immunohistochemical examination are estrogen receptor negative (ER-), progesterone receptor negative (PR-) and human epidermal growth factor receptor 2 negative (HER2-) (Dent et al., 2007; Aysola et al., 2013; Sporikova et al., 2018; Garrido-Castro et al., 2019; Yin et al., 2020). The mortality and recurrence rate of TNBC is higher than other types of breast cancer; especially in the first 5 years after diagnosis, the mortality and recurrence rate are significantly higher than other types of breast cancer (Dent et al., 2007). Primary or secondary resistance to the treatment restrained the present therapeutic strategy. Facing this grim situation, it is very urgent to identify new molecular targets to treat resistant TNBC.

Meanwhile, there is also a growing recognition of the impact of the tumor microenvironment on the fate of tumors. Tumor infiltrating immune cells (TIICs) not only affect the growth of the tumor, but also affect the effect of treatment (Ren et al., 2021; Wan et al., 2021; Zhang et al., 2021). Comprehending the interactions between cancer cells and TIICs is critical for identifying key pathogenic molecules, improving drug sensitivity, and developing new therapeutic strategies.

With the rapid development of sequencing technology, human awareness of disease has entered the genetic molecular level. Analysis of RNA sequencing (RNA-seq) data has revealed potential pathogenic genes and key molecules in various types of disease including cancer (Park, 2021; Sohrabi et al., 2021). The vigorous development of high-throughput sequencing technology and bioinformatics has provided a powerful tool for revealing the underlying molecular mechanism of breast cancer.

Here, we performed RNA sequencing of 30 pairs of tumorous tissue and matched normal tissue of 30 TNBC patients from our hospital, and conducted a combined analysis of RNA-seq data using bioinformatics methods. This study is expected to identify the potential key genes associated with tumor progression, tumor immunity and therapeutic efficacy. The findings would provide potential molecular targets for the treatment of TNBC.

## Materials and Methods

### Sample Acquisition and Pathological Diagnosis

30 pairs of cancerous tissue and matched normal tissue from 30 breast cancer patients, including 15 breast cancer with lymph node metastasis and 15 breast cancer without lymph node metastasis, were collected from our hospital. All participating patients received a standard mastectomy. During the operation, once the breast tissue was removed by the surgeon, the research technicians waiting on the side would sample part of the removed breast tissue, including the tumor and surrounding normal breast tissue. The sampled tissue would be sent for pathological examination by two professional pathologists to determine whether they were cancer or normal breast tissue. Then these diagnosed samples would be used for subsequent RNA sequencing. Written informed consent was obtained from all enrolled patients. This study was approved by the Ethics Committee of our hospital.

RNA-seq data and clinical data from the TCGA cohort of patients with breast cancer were used to investigate the survival value of key genes in the development of breast cancer.

### RNA Extraction

Total RNA was isolated and purified using TRIzol (Life, cat.265709, CA, United States) following the manufacturer’s procedure. After the quality inspection of Agilent 2,100 Bioanalyzer (Agilent, cat. G2939AA, CA, United States) and NanoPhotometer^®^ (Implen, cat. N60, Munich, Germany), mRNA with poly(A) is purified from 1 μg total RNA using VAHTS^®^ mRNA Capture Beads with Oligo (dT) (Vazyme, cat. N401-01, Nanjing, China) through two rounds of purification.

### Library Generation and RNA Sequencing

Subsequently, mRNA fragment was interrupted using VAHTS^®^ Universal V6 RNA-seq Library Prep Kit (Vazyme, cat. NR604, Nanjing, China) under 94°C 8 min and reversed transcription into cDNA which would use to synthesise U-labeled second-stranded DNAs. An A-base was added to the blunt ends of each strand to ligase the indexed adapters which contains a T-base at the tail end. After UDG enzyme treatment of the U-labeled double-strand DNA, size selection was performed with VAHTS^®^ DNA Clean Beads (Vazyme, cat. N411, Nanjing, China).

Then the ligated products are amplified with PCR by the following conditions: initial denaturation at 98°C for 5 min; 12–17 cycles of denaturation at 98°C for 10 s, annealing at 60°C for 30 s, and extension at 72°C for 30 s; final extension at 72°C for 5 min. The average insert size of cDNA library was 280 ± 80 bp. After purification by VAHTS^®^ DNA Clean Beads (Vazyme, Quality control and normalization of sequencing data cat. N411-02, Nanjing, China), quality control of concentration and fragment size is performed by Agilent 2,100 Bioanalyzer (Agilent, cat. G2939AA, CA, United States) and Qubit assay tubes (Life, cat. 1604220, CA, United States).

At last, we performed the 2 × 150 bp paired-end sequencing (PE150) on an Illumina Novaseq™ 6,000 system (Illumina Corporation, San Diego, United States) following the vendor’s recommended protocol by Guangzhou Huayin Health Medical Group CO.,Ltd. (Guangzhou, China).

### Data Quality Control and Genome Alignment

Raw reads were trimmed adapters using Cutadapt (https://cutadapt.readthedocs.io/en/stable/, v1.16) and a self-made program for removing contamination and low-quality reads which bases with a quality score lower than Q20 exceeds 50%, respectively. rRNA contamination was filtered by Bowtie2 (http://bowtie-bio.sourceforge.net/bowtie2/index.shtml, version: bowtie2, v2.3.3.1) (Langmead and Salzberg, 2012).

Clean reads were mapped to the genome GRCh37 using TopHat (http://ccb.jhu.edu/software/tophat/index.shtml,v2.1.1) (Trapnell et al., 2009). The parameters used were all default.

### Quantitation

The expression level of mRNA were calculated using RSEM (RNA-Seq by Expectation Maximization) (v1.3.1) by normalized to FPKM (Fragments Per kilobase Per Million reads) (Li and Dewey, 2011). FPKM data was further converted into TPM data for estimation of the abundance of tumor-infiltrating immune cells and for correlation analysis between interested genes and interested immune cells.

### Differential Expression Analysis

Differentially expressed genes (DEGs) were calculated with original read counts using R package “edgeR” (McCarthy et al., 2012). Note that “edgeR” is designed to work with actual read counts. Normalized data, including FPKM, RPKM and TMP, is not recommended to be used in place of actual counts in edgeR.

DEGs between 30 cancerous tissue and 30 matched normal tissue were calculated using a paired design, which can be achieved by formula of “design = model. matrix (∼patient + group)” in R. DEGs between 15 TNBC tissue with metastasis and 15 TNBC tissue without metastasis were computed using the regular analysis process.

R package “edgeR” implements novel statistical methods based on the negative binomial distribution as a model for count variability, including empirical Bayes methods, exact tests, and generalized linear models, and quasi-likelihood tests to calculate DEGs.

Prior to further analysis, “edgeR” provides a procedure to filter out low expressed genes and normalize the raw data into TMM data which is subsequently used to calculate DEGs. The R function of filterByExpr is used to filter out low expressed genes. The filterByExpr function keeps rows that have worthwhile counts in a minimum number of samples.

Selection criteria for DEGs were as follows: |logFC| > 1 and FDR <0.05. Benjamini-Hochberg method was used to adjust *p*-values.

### Functional Annotation

Functional annotation of DEGs was conducted using R package “clusterProfiler” (version: 3.18.1) (Yu et al., 2012), which provides a comprehensive set of functional annotation tools for researchers to comprehend the biological meaning behind specific gene sets. The clusterProfiler package depends on the Bioconductor annotation data GO. db and KEGG. db to obtain the maps of the entire GO and KEGG corpus. Bioconductor annotation packages org. Hs.eg.db, org. Mm.eg.db, and org. Sc.sgd.db were imported for genome-wide annotation of mapping Entrez gene identifiers or ORF identifiers for humans, mice, and yeast, respectively. Functional annotation consists of gene oncology (GO) analysis and Kyoto Encyclopedia of Genes and Genomes (KEGG) pathways, which allows one can investigate what biological functions and signaling pathways a given gene set is involved in. We also conducted gene set enrichment analysis (GSEA) based on a ranked gene set using “clusterProfiler”. GSEA could reveal some enriched signaling pathways missed in GO analysis. Key parameters were as follows: pAdjustMethod = “BH”, pvalueCutoff = 0.05, qvalueCutoff = 0.2, nPerm = 1,000, minGSSize = 10, maxGSSize = 500.

### Estimation of Tumor Infiltrating Immune Cells

Tumor-infiltrating immune cells (TIICs) were estimated using a website tool TIMER 2.0 (Newman et al., 2015; Li et al., 2020). TIMER 2.0 provides multiple computational methods based on deconvolution to characterize immune cell composition of complex tissues from their gene expression profiles. TIMER 2.0 should enable large-scale analysis of bulk RNA-seq data for cellular biomarkers and therapeutic targets. The accuracy of TIMER has been demonstrated by immunohistochemistry and flow cytometry.

### Quantification of Immune Cell Cytotoxicity

Immune cell cytotoxicity of each sample was quantified based on expression levels of *CD8A, CD8B, GZMA, GZMB* and *PRF1*, using single-sample GSEA (ssGSEA). ssGSEA is an extension of GSEA and can be used to calculate separate enrichment scores for each pairing of a sample and gene set. Each ssGSEA enrichment score represents the degree to which the genes in a particular gene set are coordinately up- or down-regulated within a sample. Key parameters were as follows: kcdf = “Gaussian”, min. sz = 1, max. sz = Inf, tau = 0.25, abs. ranking = TRUE.

### Correlation Between Hub Genes and Drug Sensitivity

Data on breast cancer cell lines were obtained from two large-scale cancer profiling studies: the Cancer Cell Line Encyclopedia (CCLE) (Barretina et al., 2012; Ghandi et al., 2019), which profiles gene expression in cancer cells, and the Cancer Therapeutics Response Portal (CTRP) (Seashore-Ludlow et al., 2015), which characterizes the response of cancer cell lines to a collection of drugs. We categorized breast cancer cell lines into low-and high-expression groups based on the median of each hug gene expression levels, and compared sensitivity to therapeutic drugs in the high-versus low-group using *t*-test. The IC50 value of each drug was used as a measure of drug response, which was available in CTRP database.

### Statistics

All statistical analyses were completed using R software (Version 4.0.1). Based on the data homogeneity of variance and normal distribution, either the independent sample *t* test or Wilcoxon signed rank test was chosen. The log-rank test was used to evaluate survival significance. Spearman’s correlation coefficient was used to assess the correlation between two continuous variables. *p* < 0.05 was considered statistically significant.

## Results

### Patient Characteristics

A total of 30 patients with diagnosed triple-negative breast cancer were included in this study, including 15 breast cancer with lymph node metastasis and 15 breast cancer without lymph node metastasis from our hospital. 30 pairs of cancerous tissue and matched normal tissue were obtained from these 30 breast cancer patients. Cancer tissue and matched normal tissue were all pathologically diagnosed by two professional pathologists to determine whether they were cancer or normal tissue. Main clinical characteristics, including age at diagnosis, tumor size, pathological grade, metastasis status and recurrence, were showed in Table 1.

**TABLE 1:** Clinical characteristics of 30 triple-negative breast cancer patients.

Patient ID	Gender	Age at diagnosis	Tumor size (cm)	Pathological grade	Lymph node	Relapse
102548	F	NA	2*1	II	yes	no
104338	F	NA	NA	II	yes	no
105094	F	NA	1*3	NA	yes	no
109745	F	NA	2.5*2.5*2	III	no	no
1906415	F	49	1.3*1.7	Ⅱ	no	yes
1912627	F	65	3.7*2.7*2.3	Ⅱ	yes	no
1924346	F	46	2*1.3	Ⅲ	no	no
1926760	F	37	4.8*2.1	III	no	no
1927842	F	36	3.6*1.3*1.5	Ⅲ	yes	yes
1933414	F	40	2.9*1.5*1.8	Ⅲ	no	no
1940640	F	66	3.1*1.2	Ⅱ	yes	no
2004407	F	64	3*2.5*1.5	III	no	no
2005288	F	46	2.5*1.8*2	Ⅲ	no	no
2006047	F	60	2.8*1.9	Ⅲ	yes	no
2008260	F	59	2*1.9	Ⅲ	no	no
2009329	F	37	2.2*1.8	Ⅲ	yes	NA
2009381	F	47	2	NA	no	no
2009850	F	49	2.6*2.4	Ⅲ	yes	no
2017611	F	57	1.7*1.2	Ⅱ	yes	no
2039179	F	42	2.3*1.7	Ⅲ	yes	no
2040686	F	40	1.7*1*1	Ⅱ	no	no
2045012	F	40	1.9*1.1	Ⅲ	no	no
2046297	F	37	5.5*5*1.2	Ⅱ	yes	no
348981	F	56	8*6*2	Ⅱ-Ⅲ	yes	no
354300	F	43	2.5*2.5*2	NA	no	no
359448	F	30	1.5	NA	no	no
94377	F	NA	2*2	II	yes	yes
98389	F	NA	2	II	no	no
98475	F	NA	7.5*2*2	NA	no	no
99145	F	NA	0.7*0.9	II	yes	no

NA, means no data is available.

### Key Genes Throughout the Oncogenesis and Progression of Breast Cancer

**FIGURE 1:**
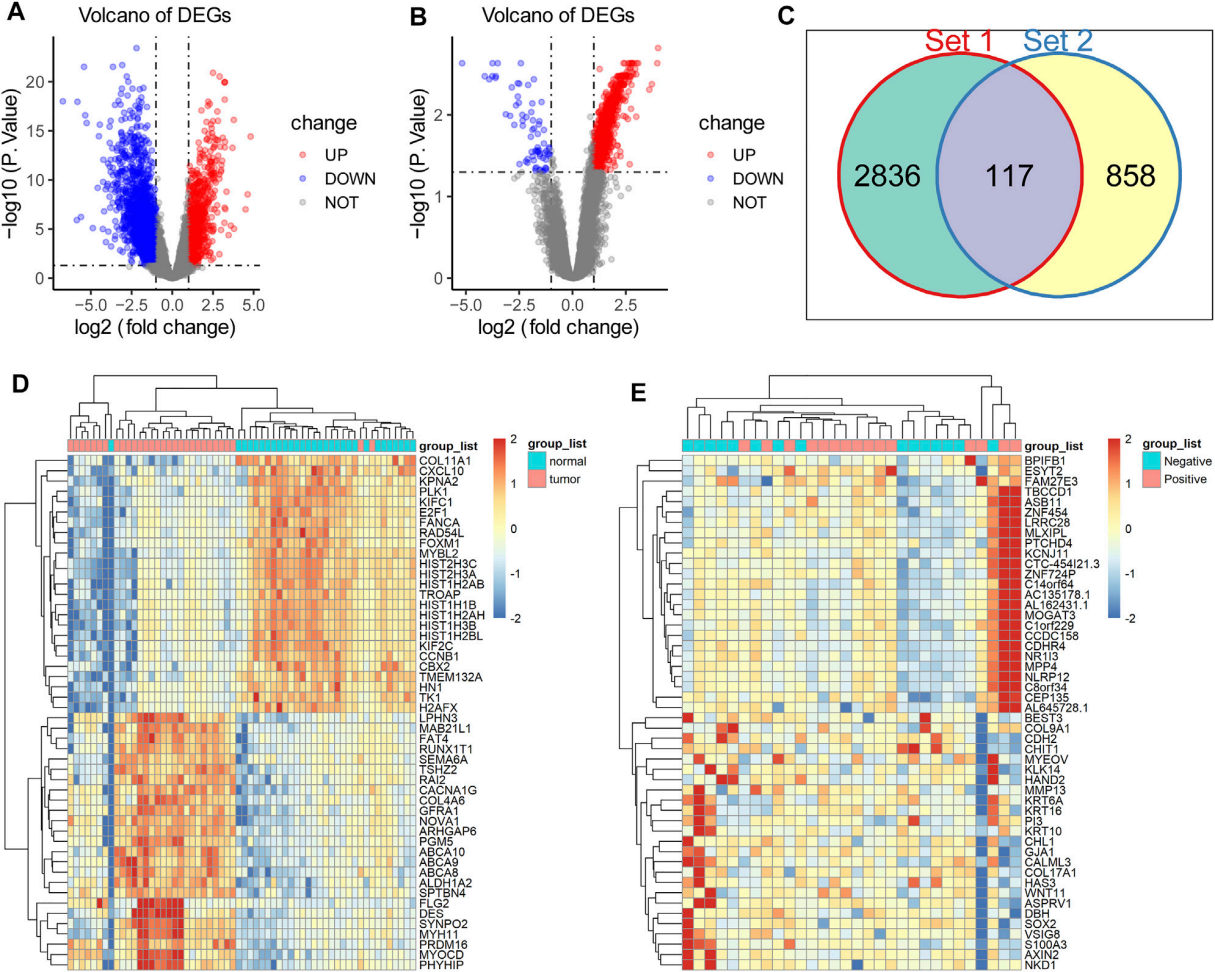
Acquisition of DEGs in TNBC. **(A)** There were 2,953 DEGs between normal and cancerous tissue, including 981 upregulated DEGs (red dots) and 1972 downregulated genes (blue dots). **(B)** There were 975 DEGs between TNBC with lymph node metastasis and TNBC without lymph node metastasis, including upregulated 905 DEGs (red dots) and 70 downregulated genes (blue dots). **(C)** There were 117 overlapping DEGs between the above two gene sets. **(D)** Heatmap anaysis showed a distinct gene expression pattern between cancerous tissue and matched normal tissue. **(E)** Heatmap analysis showed a distinct gene expression pattern between TNBC with lymph node metastasis and TNBC without lymph node metastasis.

To identify key genes throughout the oncogenesis and progression of breast cancer, we first performed differential expression analysis using RNA-seq data of 30 pairs of breast tissues (cancerous tissue and matched normal tissue) from 30 TNBC patients enrolled in this study, and then performed differential expression analysis using RNA-seq data from 15 TNBC with lymph node metastasis and 15 TNBC without lymph node metastasis. The results showed that there were 2,953 DEGs between cancerous and matched normal tissue (Figure 1A), as well as 975 DEGs between TNBC with lymph node metastasis and TNBC without lymph node metastasis (Figure 1B). There were 117 overlapping DEGs between the above two gene sets (Figure 1C), which were potential key genes involved in the oncogenesis and progression of TNBC. Meanwhile, heatmap analysis showed a distinct gene expression pattern between cancerous tissue and matched normal tissue (Figure 1D), and between TNBC with lymph node metastasis and TNBC without lymph node metastasis (Figure 1E).

### Functional Annotation of DEGs

**FIGURE 2:**
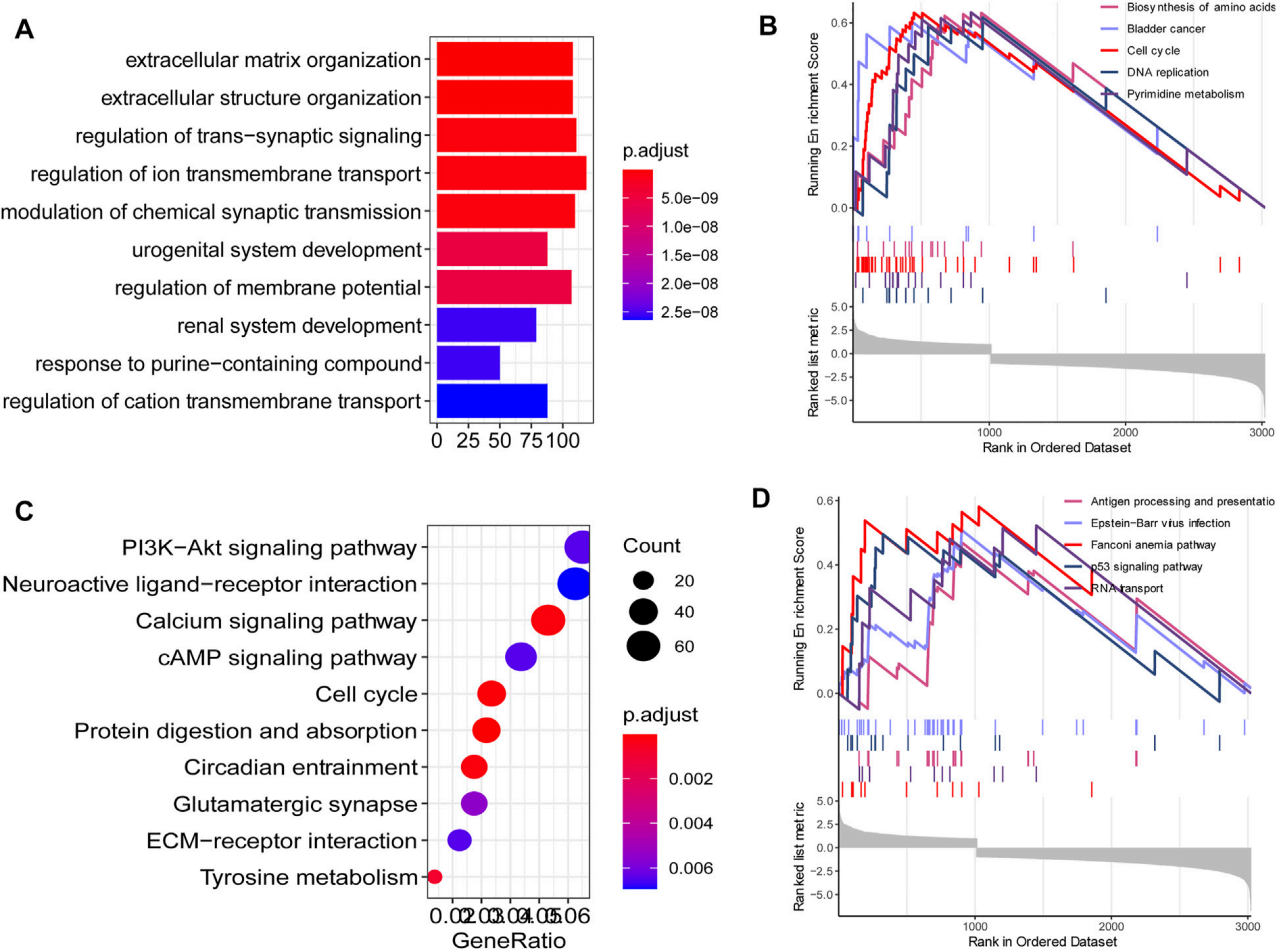
Functional annotation. **(A)** The top 10 enriched biological process in gene oncology. **(C)** The top 10 KEGG pathways in gene oncology. **(B, D)** The top 10 KEGG pathways in GSEA findings.

Since we identified 117 key DEGs throughout the development and progression of TNBC, and revealed a distinct expression present between different tissue, we next wondered to know the underlying biological function and signaling pathways. To explore the potential affected biological function and signaling pathways, we performed functional enrichment analysis for DEGs using R package “clusterProfiler”. The findings showed that extracellular matrix organization, extracellular structure organization and regulation of trans-synaptic signaling were the most enriched biological process in gene oncology (Figure 2A). PI3K-Akt signaling pathway, calcium signaling pathway, cAMP signaling pathway, and cell cycle were the most enriched KEGG pathways (Figure 2C). GSEA findings showed that cell cycle and p53 signaling pathways were the most enriched KEGG pathways (Figure 2 B, D). These signaling pathways were involved in the development of breast cancer, and potential targeted pathways in the research and treatment of breast cancer.

### Exploration of Tumor Immune Microenvironment in Breast Cancer

**FIGURE 3:**
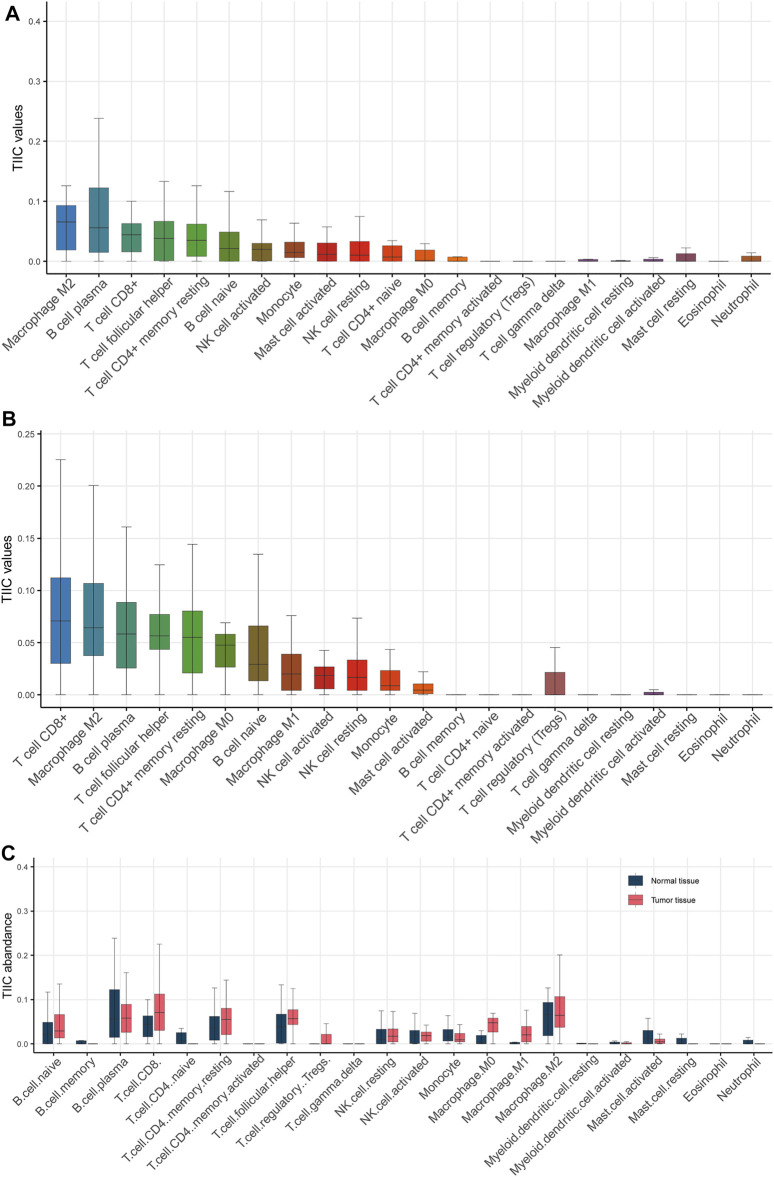
Estimation of the abundance of tumor infiltrating immune cells. **(A)** Various types of immune cells in normal tissue. **(B)** Various types of immune cells in cancerous tissue. **(C)** Boxplot showed that CD8^+^ T cell, M0 macrophage, M1 macrophage, regulatory T cell and follicular helper T cell were significantly elevated in cancerous tissue as compared with normal tissue.

Understanding the interactions between cancer and the host immune system is critical for identifying key pathogenic molecules, improving drug sensitivity, and developing new therapeutic strategies. To investigate the effects of tumor immune microenvironment on breast cancer, we estimated the abundance of immune cells in normal tissue and cancerous tissue based on RNA-seq data using TIMER 2.0. The results showed that M2 macrophage, B cell plasma and CD8^+^ T cell were the top three immune cell types in the normal tissues (Figure 3A), while CD8^+^ T cell, M2 macrophage and B cell plasma were the top three immune cell types in the cancerous tissues (Figure 3B). Notably, CD8^+^ T cell, M0 macrophage, M1 macrophage, regulatory T cell and follicular helper T cell were significantly elevated in cancerous tissue as compared with normal tissue, suggesting an elevated immune response in the tumor (Figure 3C).

### Identification of Cytotoxicity-Associated Key Genes in TNBC

Considering CD8^+^ T cell infiltration was significantly elevated in cancerous tissue, we wondered to identify key genes associated with CD8^+^ T cell infiltration. We performed a correlation analysis between the expression levels of 117 key DEGs and CD8^+^ T cell infiltration. We found there were 22 genes significantly associated with CD8^+^ T cell infiltration.

**FIGURE 4:**
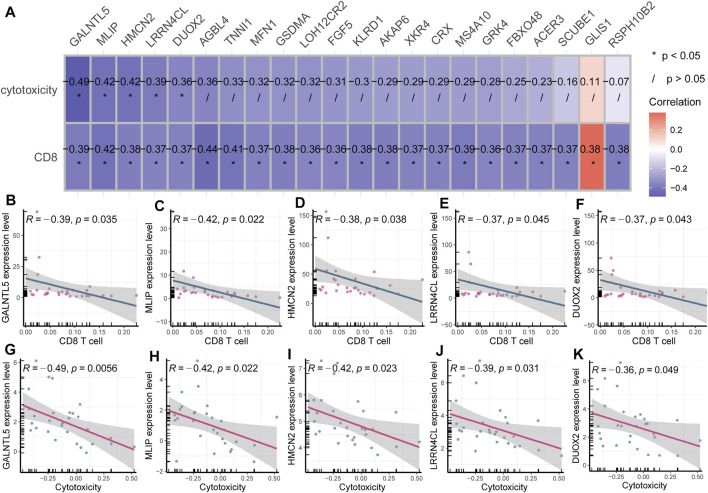
Identification of cytotoxicity-associated key genes in breast cancer **(A)** Heatmap plot displayed key genes associated with CD8^+^ T cell infiltration and cytotoxicity. **(B–F)** *GALNTL5, MLIP, HMCN2, LRRN4CL* and *DUOX2* were inversely associated with CD8^+^ T cell infiltration. **(G–K)** *GALNTL5, MLIP, HMCN2, LRRN4CL* and *DUOX2* were also negatively correlated with immune cell cytotoxicity.

To investigate whether these 22 pivotal genes were also implicated in immune cell cytotoxicity, we analyzed the association of each pivotal gene expression and immune cell cytotoxicity using Pearson correlation analysis. Immune cell cytotoxicity of each sample was quantified based on expression levels of *CD8A, CD8B, GZMA, GZMB* and *PRF1*, using ssGSEA. The expression of cytotoxicity genes *CD8A, CD8B, GZMA, GZMB* and *PRF1* can represent immune cell cytotoxicity (Huson et al., 2016; Mitchell et al., 2020) (Balint et al., 2020; Bassez et al., 2021). The results showed that five genes (*GALNTL5, MLIP, HMCN2, LRRN4CL, DUOX2*) were markedly corelated with cytotoxicity (*p* < 0.05, *R* < - 0.3; Figure 4A). These five genes were inversely associated with CD8^+^ T cell infiltration (Figures 4B–F), and also negatively correlated with cytotoxicity (Figures 4G–K), suggesting their important role in tumor immunity.

### Investigation on the Role of the Five Cytotoxicity-Associated Key Genes in TNBC

**FIGURE 5:**
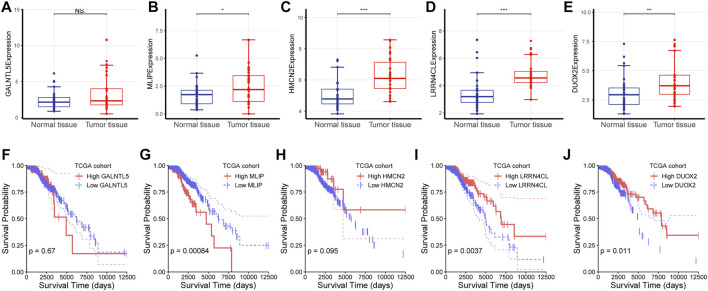
Investigation on the role of the five cytotoxicity-associated key genes **(A–E)** The expression of each gene was characterized between normal tissue and tumor tissue. NS: No significance, ∗: *p* < 0.05, ∗∗: *p* < 0.05, ∗∗∗: *p* < 0.001.

Since the above analysis revealed five key genes which were negatively correlated with CD8^+^ T cell infiltration and cytotoxicity, we wondered whether they played a role in the progression of TNBC and had a survival value. To validate their expression in the breast cancer tissue, we analyzed the expression of these five genes using RNA-seq data of breast cancer patients from our hospital. Consistent with the above findings, these four key genes (*MLIP, HMCN2, LRRN4CL*, and *DUOX2*) were critically upregulated in the cancerous tissue than in the normal tissue (t-test; *p* < 0.05; Figure 5A–E), further highlighting their protumor effects in breast cancer and their potential as a therapeutic target in cancer treatment.

Next, we analyzed the survival value of these five genes using RNAs-seq data and clinical data from the TCGA cohort of breast cancer patients, and found that *MILP*, *LRRN4CL*, and *DUOX2* had a significant survival relevance (log-rank test; *p* < 0.05), while *GALNTL5* and *HMCN2* had no survival value (log-rank test; *p* > 0.05; Figure5 F–J).

(F-J) Survival analysis was performed for *GALNTL5, MLIP, HMCN2, LRRN4CL* and *DUOX2* using log-rank test for RNA-seq data and the corresponding survival data from the TCGA cohort of patients with breast cancer.

### Association of Cytotoxicity-Associated Genes With Therapeutic Response

**FIGURE 6:**
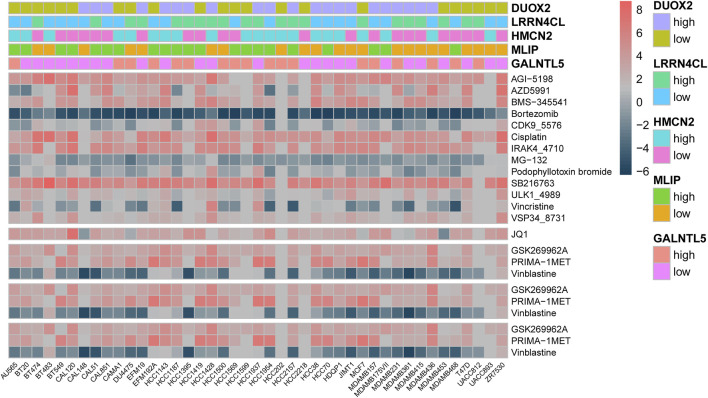
Heatmap analysis showed that *GALNTL5, MLIP, HMCN2, LRRN4CL* and *DUOX2* were associated with drug sensitivity of multiple agents in breast cancer cell lines.

As *GALNTL5, MLIP, HMCN2, LRRN4CL* and *DUOX2* were identified as cytotoxicity-associated genes, we next investigated their effects on therapeutic response by analyzing RNA-seq data and drug sensitivity of multiple breast cancer cell lines using Pearson coefficient analysis. As expected, the results showed the five cytotoxicity-associated genes were reflective of drug sensitivity of multiple agents in breast cancer cell lines (Figure 6). The targeted signaling pathways of each agent were shown in Supplementary Table S1.

## Discussion

We performed RNA sequencing for 30 pairs of TNBC tissue and matched normal tissue from our hospital, and identified five pivotal genes (*GALNTL5, MLIP, HMCN2, LRRN4CL,* and *DUOX2*), which were correlated with associated with CD8^+^ T cell infiltration, tumor progression and therapeutic efficacy. These findings will facilitate the understanding of the mechanism underlying the progression of breast cancer and the function of tumor immune microenvironment.

We observed that CD8^+^ T cell, M0 macrophage, M1 macrophage, regulatory T cell and follicular helper T cell were significantly elevated in cancerous tissue compared with normal tissue. Tumor microenvironment is a dynamic and complex system that consists of various immunocytes, including regulatory tissue-resident CD8^+^ T cells, macrophages, regulatory T cells, tumor-associated macrophages (TAMs), and so on (Chew et al., 2017). The interaction between cancerous cells and the surrounding immune cells affect the expansion, migration and invasion of tumor cells (Hernandez-Gea et al., 2013). In previous studies, CD8^+^ T cells are the predominant T cell subset in the tumor microenvironment, and correlated with improved survival outcomes in various cancers, including colorectal cancer (Mansuri et al., 2021), esophageal cancer (Hao et al., 2020), and gastric cancer (Lee et al., 2018). Macrophages play a core role in tumor immune evasion, and are expected to be the next Frontier in the immunotherapy for cancer (Qiu et al., 2021). Generally, macrophages can be divided into two categories: classically activated macrophages (M1) and alternatively activated macrophages (M2), respectively. M1 macrophages are characterized by CD68, CD86, and CD80, and secrete cytokines and chemokines like TNF-α, IL-1β, IL-12, CXCL9, CXCL10, to promote the pro-inflammatory Th1 response. M2 macrophages are featured by CD163, CD204, and CD206, and exert immunomodulatory effects, inhibiting endogenous antitumor immunity. The interaction of these tumor-infiltrating immune cells is complicated and requires to further profiling. Our findings will help to clarify the tumor microenvironment in breast cancer and the design of immunocyte-based immunotherapies.

This study still found five genes (*GALNTL5, MLIP, HMCN2, LRRN4CL, DUOX2*) associated with CD8^+^ T cell infiltration and cytotoxicity. The polypeptide N-acetylgalactosaminyltransferase-like protein 5 (GALNTL5) is involved in male fertility; however, its involvement in the development of breast cancer remains unclear (Yao et al., 2017). Muscle-enriched A-type lamin-interacting protein (MlIP) is a recently discovered Amniota gene that encodes proteins of unknown biological function (Ahmady et al., 2021). And roles of *MLIP, HMCN2* in breast cancer are unknown. *DUOX1* and *DUOX2* is an H2O2-generating enzyme related to a wide range of biological features, such as hormone synthesis, host defense, cellular proliferation, and fertilization (Fortunato et al., 2018). *DUOX1* has been involved in breast cancer, whereas the role of *DUOX2* on breast cancer is still unreported. These five newly identified genes are potential therapeutic targets in breast cancer therapy.

This study has several contributions to breast cancer research. First of all, the previously identified key biomarkers of breast cancer are either related to the pathogenesis or related to progression (Wang et al., 2021b; Dameri et al., 2021; Gonzalez-Ericsson et al., 2021; Xing et al., 2021), and few biomarkers have been identified that are related to both occurrence and progression of TNBC. Here, we identified five key genes that played a role in the pathogenesis and progression of TNBC, suggesting their potential to be candidate therapeutic targets that benefit more patients. Second, we used five key molecules to connect tumor development, tumor immunity, and drug therapy in tandem, emphasizing the central role of tumor immunity in tumor development and clinical treatment. Finally, this study included a total of 30 TNBC patients, including 30 pairs of paired RNA-seq data and corresponding detailed clinical information, which can provide research resources for others’ research.

This work has several limitations to further address. First, the study was mainly based on RNA-seq data, thereby needing further experiments *in vitro* and *in vivo*. Secondly, although we revealed the function of five key genes on CD8^+^ T cells in the tumor microenvironment, we did not explore its role in the dendritic cells and related chemokines that are involved in the function of CD8^+^ T cell.

In conclusion, we performed RNA sequencing of 30 pairs of normal and tumorous tissues from our hospital, and found several key gens associated with tumor progression and therapeutic efficacy. The findings would provide potential molecular targets for the treatment of breast cancer.

## Data Availability

The data presented in the study are deposited in the GEO repository, accession number GSE183947.
